# Perseverative Thinking and Executive Function in Adults Aged 45-70 With and Without Mood Disorders

**DOI:** 10.1093/geroni/igaf122.3276

**Published:** 2025-12-31

**Authors:** Jane Lee, Katharine Burns, Jennifer Nicoloro-Santabarbara, Faith Gunning, Katherine Burdick

**Affiliations:** Brigham and Women’s Hospital, Boston, Massachusetts, United States; Brigham and Women’s Hospital, Boston, Massachusetts, United States; Brigham and Women’s Hospital, Boston, Massachusetts, United States; Weill Cornell Medicine, New York, New York, United States; Brigham and Women’s Hospital, Boston, Massachusetts, United States

## Abstract

Emotion regulation (ER) improves with age, while executive functioning (EF) tends to decline. However, the presence of a mood disorder (MD) negatively impacts both processes. We examined whether MD diagnosis and age influence the relationship between ER and EF in 238 adults aged 45 to 70, including individuals with MD (n = 131) and healthy controls (HC; n = 107). ER was assessed using the Perseverative Thinking Questionnaire (PTQ) and an EF composite score was derived from four cognitive tasks. Participants were grouped into three age groups: 45–53 (n = 72), 54–62 (n = 102), and 63–70 (n = 64). Hierarchical regression analysis revealed a main effect of EF on PTQ (β=-0.264, p < 0.05), though all interaction terms were non-significant. Follow-up correlational analysis showed that EF was negatively associated with PTQ in middle-aged HCs only (r =-.437, p < 0.05). ANOVA results revealed significant age-related differences in the PTQ within the MD group (F(2, 119), 3.769, p<.05). Tukey post hoc comparisons indicated that younger individuals with MD exhibited higher levels of perseverative thinking (PT) compared to older individuals (p < 0.05). These findings suggest that in middle-aged HCs, EF is protective from PT, a form of maladaptive ER. However, in MD, EF was not significantly correlated with PT across age groups, and younger individuals with MD exhibited higher PT than older individuals. This suggests that factors beyond EF contribute to PT in individuals with MD. Therefore, interventions targeting individuals with MD should address both cognitive and emotional factors contributing to PT.

